# Social disadvantage and the black-white disparity in spontaneous preterm delivery among California births

**DOI:** 10.1371/journal.pone.0182862

**Published:** 2017-08-11

**Authors:** Suzan L. Carmichael, Peiyi Kan, Amy M. Padula, David H. Rehkopf, John W. Oehlert, Jonathan A. Mayo, Ann M. Weber, Paul H. Wise, Gary M. Shaw, David K. Stevenson

**Affiliations:** 1 Division of Neonatal and Developmental Medicine, Department of Pediatrics, Stanford University School of Medicine, Stanford, CA, United States of America; 2 Division of Maternal-Fetal Medicine, University of California San Francisco School of Medicine, San Francisco, CA, United States of America; 3 Division of General Medical Disciplines, Department of Medicine, Stanford University School of Medicine, Stanford, CA, United States of America; University of Bristol, UNITED KINGDOM

## Abstract

We examined the contribution of social disadvantage to the black-white disparity in preterm birth. Analyses included linked vital and hospital discharge records from 127,358 black and 615,721 white singleton California births from 2007–11. Odds ratios (OR) were estimated by 4 logistic regression models for 2 outcomes: early (<32 wks) and moderate (32–36 wks) spontaneous preterm birth (ePTB, mPTB), stratified by 2 race-ethnicity groups (blacks and whites). We then conducted a potential impact analysis. The OR for less than high school education (vs. college degree) was 1.8 (95% confidence interval 1.6, 2.1) for ePTB among whites but smaller for the other 3 outcome groups (ORs 1.3–1.4). For all 4 groups, higher census tract poverty was associated with increased odds (ORs 1.03–1.05 per 9% change in poverty). Associations were less noteworthy for the other variables (payer, and tract percent black and Gini index of income inequality). Setting 3 factors (education, poverty, payer) to ‘favorable’ values was associated with lower predicted probability of ePTB (25% lower among blacks, 31% among whites) but a 9% higher disparity, compared to probabilities based on observed values; for mPTB, respective percentages were 28% and 13% lower probability, and 17% lower disparity. Results suggest that social determinants contribute to preterm delivery and its disparities, and that future studies should focus on ePTB and more specific factors related to social circumstances.

## Introduction

Preterm delivery (i.e., delivery at <37 weeks gestation) affects approximately 11% of U.S.-born infants and is one of the most common causes of infant morbidity and mortality [1]. Babies born to black mothers have a prevalence of preterm delivery that is twice that of infants born to white mothers. One potential contributor to this disparity is social disadvantage, which is much more prevalent among blacks than whites and associated with higher risk of preterm delivery. Its actual contribution to the disparity is unclear. Several studies report that after adjustment for markers of social disadvantage, the disparity changes minimally [2–4]. Others suggest the disparity is greatest among the most socially *advantaged* women [2, 3, 5].

A fundamental challenge to understanding the contribution of social disadvantage to this disparity is that social disadvantage is not a singular construct. Markers related to education and income are most commonly examined; others are potentially of importance but less frequently examined, such as segregation and inequality [6–9]. Social disadvantage may incur higher risk through many pathways, such as reduced access to care, worse nutrition, increased stress, higher-risk reproductive patterns (e.g., teen birth, high parity) and higher prevalence of conditions such as obesity, diabetes and hypertension. Studies vary in how they deal with these potential pathways; many adjust for variables along the pathways, but this approach may result in over-adjustment and thus an underestimate of the contribution of social disadvantage to disparities. Another challenge is structural confounding, unless very large populations are available for study [5]. In addition, preterm deliveries are usually examined as a single group, despite evidence of etiologic heterogeneity based on timing of delivery and events that instigated delivery, e.g., how early the delivery was and whether it was spontaneous or induced [6]. Sample sizes are often not large enough and/or clinical data are not available to enable these distinctions.

Our objective was to investigate the contribution of multiple individual- and residential area- level factors related to social disadvantage to the black-white disparity in preterm delivery among a large population of California births, which represent one in eight of all U.S. births. We addressed the presence of effect modification among individual- and area- level factors, as well as whether their associations with preterm delivery differed for blacks versus whites. We separately examined early (i.e., <32 weeks) and moderate (i.e., 32–36 week) preterm deliveries and focused on those that were spontaneous (i.e., preceded by spontaneous onset of labor or rupture of membranes). We focused on spontaneous preterm deliveries because their etiology may be distinct from those that are medically indicated, and they comprise the vast majority of preterm deliveries in this dataset (86% of early preterm deliveries and 72% of moderate preterm deliveries).

In addition to examining risks, we conducted a potential impact analysis to consider how different the disparity might be if social factors were more equal between blacks and whites. That is, we used a substitution estimator approach to gauge the potential impact of a counterfactual change in social disadvantage on the prevalence of spontaneous preterm delivery and the black-white disparity [10, 11].

## Methods

The study population included 822,414 singleton infants born to non-Hispanic black and white mothers in California from 2007–2011 (referred to hereafter as ‘black’ and ‘white’ infants) and whose birth certificates were successfully linked to their delivery hospital discharge records by the Office of Statewide Health Planning and Development (>98% of births).

Individual-level markers of social disadvantage included maternal education (less than high school, equal to high school, some college, college degree) and payer for the delivery hospitalization (Medi-Cal, private, uninsured, other).

To create census tract variables, we geocoded maternal residential addresses at delivery, which we obtained from electronic birth certificates, and then assigned one of California’s >8,000 census tracts (using PROC GEOCODE, SAS 9.4, U.S. Census 2015 TIGER/Line^R^ Shapefiles, up to 4 iterations). Geocoding was successful for 782,861 subjects (95.2%).

We examined three census tract-level markers of social disadvantage, which we derived from 2007–2011 American Community Survey files: poverty as measured by percent of the tract population with household income below the poverty level; percent of the tract population that was black, as a basic measure of segregation; and the Gini index of income inequality, a measure of census tract income distribution calculated by the Census Bureau (0 reflects a completely proportional distribution of income, 1 reflects one person having all the income). In addition to poverty, we created an index that incorporated eight census tract variables representing multiple aspects of socioeconomic level (poverty, occupation, employment, education, and housing) following previously described methods [12]. Its correlation with tract poverty was high (r = 0.86). We therefore included poverty rather than the index in our main models [13–15].

Gestational age was based on best obstetric estimate from birth certificates. We excluded 11,160 infants with gestational age that was missing or outside 20–41 weeks and then 15,041 with any other missing variables, leaving 756,660 births (625,778 white, 130,882 black) available for analysis, with no missing data on covariates. The outcome of interest was early (20–31 weeks) or moderate (32–36 weeks) spontaneous preterm delivery, i.e., those preceded by preterm premature rupture of membranes (ICD-9-CM code 658.1 or birth certificate complication of labor/delivery code 10), premature labor (ICD-9-CM code 644), or use of tocolytics (birth certificate complication/procedure of pregnancy code 28). Other preterm deliveries were induced or delivered by cesarean section without a code for spontaneous onset of labor (medically indicated). These types of variables have been shown to have good validity in administrative hospital discharge data [16].

We used logistic regression to estimate odds ratios (OR) and 95% confidence intervals (CI) for early and moderate spontaneous preterm delivery. The reference group was term infants delivered at 37–41 weeks. Non-spontaneous preterm deliveries were excluded (10,057 whites, 3,524 blacks). Initial models included maternal black-white race-ethnicity, education, payer, and census tract poverty, percent black, and Gini index (tract-level variables were specified as continuous). We restricted the model to these variables given our objective of examining the total contribution of social disadvantage to preterm delivery and the premise that social disadvantage leads to preterm delivery via many pathways. These pathways include maternal reproductive patterns, which to some extent drive the demographics of women who deliver, and thus we did not adjust for such factors (e.g., age, parity). We tested the interaction of individual and census tract socioeconomic level (i.e., maternal education and tract poverty) and of black-white race-ethnicity with maternal education and the three tract-level variables (poverty, Gini index, percent black) (i.e., 5 interactions in total, per model), inputting one interaction (as a product term) at a time into each baseline model. For early preterm delivery, all five interaction terms had P<0.10. For moderate preterm delivery, only education by poverty and black-white race-ethnicity by poverty had P<0.10. Based on the multiple significant interactions with black-white race-ethnicity, we ran further models separately for blacks and whites. Within these stratified models, the interaction of education by poverty was not significant for early preterm delivery for blacks or whites (P>0.10) and was therefore not included in subsequent models. We used conventional logistic regression due to its relative simplicity, its amenability to our initial tests of interaction, and minimal concern about independence of observations since there are >8,000 census tracts in California.

We then conducted a potential impact analysis to consider what the prevalence of spontaneous preterm delivery and the black-white disparity might be in the hypothetical situation of a similar level of social disadvantage among blacks and whites. To do this, we followed substitution estimator methods described by Ahern et al. to estimate the unobserved counterfactual probability of preterm delivery at specific imputed levels of education, payer status and poverty [10, 11]. We did not estimate impact for percent black or the Gini index because most confidence intervals for these variables included 1.0.

First, we estimated predicted probabilities of spontaneous preterm delivery for each *individual*, for each specified scenario. We did this by using coefficients from our final logistic regression models to estimate the predicted log odds (plo_x_) for each individual at different values of the specified variables, and the following equation to estimate the predicted probability: PProb_x_ = 1/(1+exp(-1 x plo_x_)) where *x* refers to the value(s) of the variable(s) we manipulated. As our baseline comparator, we estimated the expected probability of preterm delivery after inputting each mother’s *observed* values of all variables. We then estimated counterfactual probabilities after substituting values for the predictor variables to reflect each following scenario, for all subjects: 1) input education to correspond to each of its four levels (with age-specific exceptions described below); 2) input payer to each of its four levels; 3) input census tract poverty to range from 2% to 42%, to reflect its observed range (the 1 percentile values for poverty were 2% among blacks and 1% among whites; the respective 99 percentile values were 57% and 42%); 4) input all 3 variables as favorable (i.e., education as college degree, payer as private, poverty as 2%); 5) input all 3 variables as unfavorable (i.e., education as less than high school, payer as Medi-Cal, poverty as 42%). We chose Medi-Cal as the value for the unfavorable scenario because eligibility is income-based, and it is much more common than uninsured payer.

Given that educational potential varies by age, we made the following exceptions in assigning imputed education values. For women <18 years old, the maximum substituted value was ‘less than high school’; for women 18 years old, it was ‘equal to high school;’ and for women 19–21 years old, it was ‘some college’. For example, for women <18 years old, if the intended substituted value was high school education, and her observed value was less than high school, we retained her observed value.

Second, we estimated the *overall* predicted probability (PProb) of spontaneous preterm delivery among all black and white women, and the black-white disparity (i.e., the ratio of the PProb for blacks and whites), for each counterfactual scenario; as well as the percent change in the PProb and disparity, relative to those based on observed values, for each scenario. For each PProb, percent change in PProb, black-white disparity, and percent change in disparity, we estimated confidence intervals using a nonparametric bootstrap [10]. This study is approved by the California Committee for the Protection of Human Subjects and the Stanford University Institutional Review Board.

## Results

The prevalence of preterm delivery among study subjects was 10.2% among blacks and 6.3% among whites (Table 1). The prevalence of early spontaneous preterm delivery was 1.8% among blacks (n = 2,390) and 0.6% among whites (n = 4,019), giving an unadjusted prevalence ratio of 3.0 (95% CI 2.8, 3.1). The prevalence of moderate spontaneous preterm delivery was 5.7% among blacks (n = 7,489) and 4.1% (n = 25,388) among whites, giving an unadjusted prevalence ratio of 1.5 (95% CI 1.4, 1.5). A total of 2.7% of blacks (n = 3,524) and 1.6% of whites (n = 10,057) had preterm deliveries that were medically indicated or of unknown subtype. Black mothers were more likely than white mothers to have less than high school education (17% vs. 6%) and less likely to have a college degree or higher (14% vs. 44%), and they were more likely to have Medi-Cal (55% vs. 23%) and less likely to have private insurance (37% vs. 72%) (Table 1). The median percent of the census tract population living below the poverty level was 19% for blacks and 9% for whites; the median percent tract population that was black was 14% for blacks and 2% for whites.

**Table 1:** Prevalence of preterm delivery and descriptors of singleton infants born to non-hispanic black and white mothers in California, 2007–2011.

	Infants born to black mothers (n = 130,882)	Infants born to white mothers (n = 625,778)
**Prevalence of preterm delivery (per 100 births):**	Percent	Percent
Early spontaneous preterm delivery (20–31 weeks)	1.8	0.6
Moderate spontaneous preterm delivery (32–36 weeks)	5.7	4.1
Preterm delivery that was medically indicated or unknown subtype	2.7	1.6
Total prevalence	10.2	6.3
**Individual-level descriptors:**		
Maternal age at delivery		
<20 years	13.6	4.3
20–35 years	76.0	77.9
>35 years	10.3	17.8
Maternal nulliparity	41.7	44.9
Maternal education		
Less than high school	16.7	6.1
Equal to high school	34.5	21.7
Some college	34.9	28.6
College degree or higher	13.9	43.6
Payment for delivery		
Medi-Cal	55.0	23.3
Private	36.6	72.0
Uninsured	2.2	1.2
Other	6.2	3.5
Census tract-level descriptors:*	Median (25th-75th percentile)	Median (25th-75th percentile)
Percent of population with income below poverty level	18.6 (10.6–28.6)	9.2 (5.3–15.7)
Gini index of income inequality	0.42 (0.38–0.48)	0.44 (0.38–0.69)
Percent of population that is black	13.9 (6.2–25.8)	2.3 (0.8–5.7)

Table 2 provides results for multivariable logistic regression models for early and moderate spontaneous preterm delivery. Among black women, education less than a college degree was associated with 23–32% increased odds of preterm delivery. Among white women, education was associated with 43–83% increased odds of early preterm delivery, increasing monotonically with lower education; associations with moderate preterm delivery were more modest (17 to 37% increase). Relative to private insurance, being uninsured was associated with increased odds of early (OR 3.07 for blacks, 95% CI 2.55, 3.69, and 3.73 for whites, 95% CI 3.18, 4.37) and moderate preterm delivery (OR 2.10 for blacks, 95% CI 1.85, 2.39, and 2.09 for whites, 95% CI 1.92, 2.28), but only 2% of black women and 1% of white women were uninsured. ORs for Medi-Cal and other insurance were smaller, ranging from 0.78 to 1.17. For all four groups, higher census tract poverty was associated with increased odds (3–5% increased odds per 9% change in poverty). For census tract percent black, the ORs per 6% higher percent of blacks ranged from 1.00 to 1.03 across the four models. For the Gini index, the ORs per 0.1-unit change ranged from 0.99 to 1.01.

**Table 2:** Association of markers of social disadvantage with odds of spontaneous early (<32 weeks) and moderate (32–36 weeks) preterm delivery, relative to term delivery (37–41 weeks).^a^

	Adjusted odds ratio (95% CI) for early preterm delivery		Adjusted odds ratio (95% CI) for moderate preterm delivery	
	Blacks	Whites	Blacks	Whites
Maternal education				
Less than high school	1.29 (1.09, 1.52)	1.83 (1.61, 2.08)	1.32 (1.20, 1.45)	1.37 (1.30, 1.45)
Equal to high school	1.23 (1.06, 1.42)	1.46 (1.34, 1.60)	1.20 (1.10, 1.31)	1.14 (1.10, 1.18)
Some college	1.24 (1.08, 1.43)	1.43 (1.32, 1.54)	1.23 (1.13, 1.34)	1.17 (1.13, 1.20)
College degree or higher	reference	reference	reference	reference
Payment for Delivery				
Medi-Cal	0.94 (0.86, 1.04)	1.17 (1.08, 1.27)	1.07 (1.01, 1.13)	1.04 (1.01, 1.08)
Private	reference	reference	reference	reference
Uninsured	3.07 (2.55, 3.69)	3.73 (3.18, 4.37)	2.10 (1.85, 2.39)	2.09 (1.92, 2.28)
Other	0.78 (0.65, 0.95)	1.04 (0.88, 1.23)	1.07 (0.96, 1.19)	1.03 (0.96, 1.10)
Census tract poverty: OR per 1% change and 9% change ^b^	1.005 (1.002, 1.008) 1.05 (1.02, 1.08)	1.004 (1.001, 1.008) 1.04 (1.01, 1.07)	1.005 (1.003, 1.007) 1.05 (1.03, 1.06)	1.003 (1.001, 1.004) 1.03 (1.01, 1.04)
Census tract Gini index of income inequality: OR per 0.1-unit change	1.01 0.99, 1.03)	0.99 (0.98, 1.00)	0.99 (0.98, 1.01)	1.00 (0.99, 1.00)
Census tract percent black: OR per 1% change and 6% change ^b^	1.002 (0.999, 1.004) 1.01 (1.00, 1.02)	1.006 (1.001, 1.010) 1.03 (1.00, 1.06)	1.000 (0.999, 1.001) 1.00 (0.99, 1.01)	1.000 (0.998, 1.002) 1.00 (0.99, 1.01)

^a^ All variables were included in the models, which included 2,390 early preterm, 7,489 moderate preterm, and 117,479 term deliveries to black women and 4,019 early preterm, 25,388 moderate preterm, and 586,314 term deliveries to white women.

^b^ In addition to ORs associated with a 1% change in census tract poverty and percent blacks, we present ORs for a 9% change for poverty and a 6% change in percent blacks (the SDs for poverty and percent blacks were 9% and 6% for whites; they were 13% and 18% for blacks).

Table 3 provides results examining the predicted probability (PProb) of early and moderate spontaneous preterm delivery and the black-white disparity, based on observed and counterfactual (substituted) values of education, payer and census tract poverty. When we counterfactually set education to college degree, the PProb of early preterm delivery was 11.9% lower among blacks and 18.3% lower among whites, and the black-white disparity was 7.8% higher, as compared to values obtained when incorporating observed values of education (as well as all the other variables). In contrast, when we set education equal to high school, the respective probabilities were 6.3% and 41.8% higher and the disparity was 25.1% lower as compared to estimates based observed education. Setting everyone to private insurance was associated with an 8.7% increase in the disparity in early preterm delivery, whereas setting everyone to Medi-Cal was associated with a 12.3% decrease; for moderate preterm delivery, the respective percentages were 3.6% and 1.3% decreases in the disparity. Setting payer to uninsured was associated with much larger increases in the PProb (86.1–237.5%), and reductions in the black-white disparity (12.6% for early and 4.9% for moderate preterm). Changing poverty was associated with modest changes in the disparity. Setting all three factors to ‘favorable’ values was associated with substantially lower PProb of early preterm delivery (24.6% lower among blacks, 30.7% among whites) but a 8.8% higher disparity, as compared to the PProb for observed values of the three factors. Setting all three to ‘unfavorable’ values was associated with higher probability of early preterm delivery (11.2% higher among blacks, 75.2% among whites) and a 36.6% lower disparity. For moderate preterm delivery, setting all three factors to favorable or unfavorable values was associated with a lower disparity (16.8% and 11.5%, respectively).

**Table 3:** Predicted probability (PProb) of early and moderate spontaneous preterm delivery (per 100 births) among blacks and whites and the black-white disparity, based on observed and counterfactual (substituted) values of social disadvantage variables, and the percent change in the PProb and disparity relative to observed values, California singleton births, 2007–2011. ^a^

Inputs into the models	Early preterm delivery						Moderate preterm delivery					
	Black		White				Black		White			
	PProb (95% CI)	% Change (95% CI)	PProb (95% CI)	% Change (95% CI)	Disp. (95% CI)	% Change (95% CI)	PProb (95% CI)	% Change(95% CI)	PProb (95% CI)	% Change(95% CI)	Disp. (95% CI)	% Change (95% CI)
Observed values of explanatory variables (no substitution):	2.0	–	0.7	–	2.9	–	6.0	–	4.2	–	1.5	–
	(2.0–2.0)	–	(0.7–0.7)	–	(2.9–2.9)	–	(6.0–6.0)	–	(4.2–4.2)	–	(1.4–1.5)	–
Substituted values of explanatory variables:												
Education:												
Less than high school	2.1	6.3%	1.0	41.8%	2.2	-25.1%	6.5	8.8%	5.1	23.2%	1.3	-11.7%
	(2.1–2.1)	(6.1–6.4)	(1.0–1.0)	(41.7–41.9)	(2.2–2.2)	(-25.2- -24.9)	(6.5–6.5)	(8.7–8.9)	(5.1–5.1)	(23.1–23.2)	(1.3–1.3)	(-11.7- -11.6)
High school	2.0	1.6%	0.8	13.8%	2.6	-10.8%	6.0	0.04%	4.3	3.4%	1.4	-3.2%
	(2.0–2.0)	(1.4- 1.8)	(0.8–0.8)	(13.8–13.9)	(2.6–2.6)	(-10.9- -10.6)	(6.0–6.0)	(-0.03–0.1)	(4.3–4.3)	(3.4- 3.4)	(1.4–1.4)	(-3.3- -3.2)
Some college	2.1	2.4%	0.8	11.0%	2.7	-7.7%	6.1	2.2%	4.4	5.7%	1.4	-3.3%
	(2.1–2.1)	(2.2–2.6)	(0.8–0.8)	(10.9–11.0)	(2.7–2.7)	(-7.9- -7.6)	(6.1–6.1)	(2.1- 2.3)	(4.4–4.4)	(5.7- 5.7)	(1.4–1.4)	(-3.4- -3.3)
College degree	1.8	-11.9%	0.6	-18.3%	3.2	7.8%	5.3	-11.1%	3.9	-7.2%	1.4	-4.1%
	(1.8–1.8)	(-12.1- -11.8)	(0.6–0.6)	(-18.4- -18.3)	(3.2–3.2)	(7.7–8.0)	(5.3–5.4)	(-11.1- -11.0)	(3.9–3.9)	(-7.3- -7.2)	(1.4–1.4)	(-4.2- -4.0)
Payer status:												
Medi-Cal	1.9	-5.4%	0.7	7.8%	2.6	-12.3%	6.0	0.1%	4.2	1.5%	1.4	-1.3%
	(1.9–1.9)	(-5.5- -5.4)	(0.7–0.7)	(7.7–7.8)	(2.6–2.6)	(-12.3- -12.2)	(6.0–6.0)	(0.1–0.2)	(4.2–4.2)	(1.4–1.5)	(1.4–1.4)	(-1.4- -1.3)
Private	2.0	0.1%	0.6	-7.9%	3.2	8.7%	5.7	-5.9%	4.1	-2.3%	1.4	-3.6%
	(2.0–2.0)	(0.1–0.2)	(0.6–0.6)	(-7.9- -7.2)	(3.2–3.2)	(8.6–8.7)	(5.7–5.7)	(-5.9- -5.8)	(4.1–4.1)	(-2.3- -2.3)	(1.4–1.4)	(-3.7- -3.6)
Uninsured	5.9	195.0%	2.3	237.5%	2.6	-12.6%	11.2	86.1%	8.1	95.7%	1.4	-4.9%
	(5.9–5.9)	(194.9–195.2)	(2.3–2.3)	(237.3–237.6)	(2.6–2.6)	(-12.6- -12.5)	(11.2–11.2)	(86.0–86.2)	(8.1–8.1)	(95.7–95.8)	(1.4–1.4)	(-5.0- -4.9)
Other	1.6	-21.1%	0.7	-4.1%	2.4	-17.8%	6.0	0.2%	4.2	0.4%	1.4	-0.2%
	(1.6–1.6)	(-21.2- -21.1)	(0.7–0.7)	(-4.1- -4.0)	(2.4–2.4)	(-17.8- -17.8)	(6.2–6.0)	(0.2–0.3)	(4.2–4.2)	(0.4- 0.5)	(1.4–1.4)	(-0.2- -0.1)
Census tract poverty: ^b^												
2%	1.8	-9.1%	0.7	-4.5%	2.8	-4.8%	5.5	-8.6%	4.0	-2.7%	1.4	-6.1%
	(1.8–1.8)	(-9.2- -8.9)	(0.7–0.7)	(-4.6- -4.4)	(2.8–2.8)	(-4.9- -4.6)	(5.5–5.5)	(-8.7- -8.5)	(4.0–4.0)	(-2.8- -2.7)	(1.4–1.4)	(-6.2- -6.0)
42%	2.2	10.6%	0.8	13.2%	2.9	-2.4%	6.6	9.9%	4.5	8.4%	1.5	1.4%
	(2.2–2.2)	(10.4–10.7)	(0.8–0.8)	(13.1–13.3)	(2.9–2.9)	(-2.5- -2.2)	(6.6–6.6)	(9.8–10.0)	(4.5–4.5)	(8.4–8.5)	(1.5–1.5)	(1.3–1.5)
Substituted values of all 3 variables: ^c^												
All favorable	1.5	-24.6%	0.5	-30.7%	3.2	8.8%	4.3	-27.8%	3.6	-13.2%	1.2	-16.8%
	(1.5–1.5)	(-24.6- -24.6)	(0.5–0.5)	(-30.7- -30.7)	(3.2–3.2)	(8.8–8.8)	(4.3–4.3)	(-27.8- -27.7)	(3.6–3.6)	(-13.2- -13.2)	(1.2–1.2)	(-16.8- -16.8)
All unfavorable	2.2	11.2%	1.2	75.2%	1.9	-36.6%	7.2	20.0%	5.6	35.6%	1.3	-11.5%
	(2.2–2.2)	(11.1–11.2)	(1.2–1.2)	(75.2–75.2)	(1.9–1.9)	(-36.6- -36.6)	(7.2–7.2)	(20.0–20.0)	(5.6–5.6)	(35.6–35.6)	(1.3–1.3)	(-11.5- -11.5)

^a^ All estimates are derived from separate logistic regression models for blacks and whites and early and moderate spontaneous preterm delivery that included maternal education, payer for delivery hospitalization, and census tract poverty, percent black, and Gini index (results from those models are presented in Table 2). For counterfactual estimates, all subjects were set to the same value of the specified variable(s), with some exceptions for education to account for maternal age (see Methods for more detail). Percent change reflects percent change in predicted probabilities and disparities when using counterfactual (substituted) versus observed values of the variables.

^b^ Poverty was set to 2%, which corresponds to the 1 percentile value for blacks and 5 percentile for whites or 42%, which corresponds to the 99th percentile for whites and the 95^th^ for blacks.

^c^ All favorable: set education to college degree, payer to private, and poverty to 2%. All unfavorable: set education to less than high school, payer to Medi-Cal, and poverty to 42%.

Confidence intervals for all of the point estimates except one in Table 3 excluded the null value, and they tended to be very narrow (most upper and lower limits deviated less than +/- 0.10 from their respective point estimates).

## Discussion

In this study of California births, the risk of early spontaneous preterm delivery was 3-fold higher among black than white infants. The risk for moderate spontaneous preterm delivery was 1.4-fold higher. With a few exceptions, the contribution of markers of social disadvantage to odds of spontaneous preterm delivery among blacks and whites and the black-white disparity tended to be modest, as evidenced by logistic regression models and a potential impact analysis.

Social disadvantage is much more prevalent among blacks than whites. In our study population, 14% of black but 44% of white mothers had a college degree; 37% of black but 72% of white mothers had private health insurance; and black mothers lived in census tracts with a much higher prevalence of poverty. Many studies have investigated the extent to which these types of variables may explain the higher prevalence of preterm delivery among blacks. Results have been mixed but in general suggest that the disparity is not easily explained by them [3, 17, 18]. Our results concur, even with the inclusion of varied measures of social disadvantage and more focused phenotypes. As an example, our potential impact analysis suggests that even if we set multiple social disadvantage variables to ‘favorable’ values for everyone, the majority of the variability in the black-white disparity is not explained. In fact, under this scenario, we estimate that the disparity in early preterm delivery would actually increase by 8.8%, whereas the disparity in moderate preterm delivery would decrease by 16.8%.

Given the stronger disparity for early than moderate preterm delivery and some differences in results for early and moderate preterm delivery, we recommend that future studies differentiate between these subgroups. A focus on early preterm delivery is particularly important, given its stronger disparity, associated morbidity, and less frequent study. Although prior research suggests that associations with some risk factors may be stronger for earlier than later preterm deliveries and vary for spontaneous versus medically indicated births [18], most prior studies examine all preterm deliveries together. As noted above, we focused on spontaneous preterm deliveries because their etiology may be distinct and they comprise most preterm deliveries in this dataset; future studies of medically indicated preterm delivery are needed. In addition, most prior studies focus on indicators of socioeconomic level, whereas we also included measures of segregation and inequality. These latter measures did not however contribute substantially to risk. Some studies have suggested they contribute, but study designs and settings have varied widely [2, 7, 9, 19–21]. However, each of the measures in our study is relatively general, and more in-depth study would be informative. Further studies could include more complex and multi-level measures of segregation and inequality [7, 8, 22] and consider factors associated with social disadvantage that may have a more direct impact on health risks such as health care access and quality, stress-associated conditions such as crime, environmental exposures, and pre- existing maternal medical conditions. Studies of racism against blacks would also likely be informative. We hope our results will serve as a springboard for such analyses in the future.

We used results from logistic regression models, which emphasize individual-level estimates, to conduct a potential impact analysis, which emphasizes population-level estimates. We do not consider observed associations to be directly causal but rather consider the impact analysis to be a thought experiment to gauge the potential contribution of social disadvantage to population-level prevalence. Prior studies of preterm delivery have not typically explored such estimates, but extensive justification exists for doing so, as long as results are interpreted carefully [23–25]. Prior studies have used various approaches to estimate the extent to which health outcomes and disparities may be attributable to social factors [26, 27]. We used a substitution estimator approach [10, 11], which has the advantages of being based on individual-level estimates, allowing incorporation of multiple covariates and interactions, and enabling manipulation of multiple variables at a time. Given how prevalent social disadvantage is, especially among blacks, even modest associations have the potential to explain a substantial proportion of risk and disparity.

The extent to which the probability of preterm delivery and its black-white disparity changed under different scenarios of the potential impact analysis varied, and not always in ‘favorable’ directions. Substitution of most of the study variables with a constant value resulted in modest predicted change in the black-white disparity (<15% for most scenarios), and the predicted change was usually a decrease in the disparity. Notably, substituting education as college degree and payer as private (one at a time, or together, while also changing tract-level poverty to low) resulted in a predicted *increase* in the disparity for early preterm birth, by 8–9% for each scenario. In addition, some changes were more dramatic; e.g., the probability of early preterm delivery among whites was predicted to increase 75.2% after substituting ‘unfavorable’ values for multiple variables, but only 11.2% among blacks. This variability in results stems from a combination of the odds ratios and actual distribution of each variable, and how different they were between blacks and whites. We chose extremes for illustration (e.g., everyone living in tracts with <2% poverty, everyone having less than high school education) not because we think they are feasible (or in some cases desirable) but rather to illustrate the maximum amount of change that could result, and in what direction, given the strength of the associations estimated by the logistic regression models and varied distributions of the predictor variables among blacks and whites. Results from the impact analysis, and in particular estimates that are based on setting all variables to ‘favorable’ or ‘unfavorable,’ provide perspective on the proportion of PTB and its black-white disparity that may be attributable to these types of variables.

Strengths of our study include its population-based design, large sample size which enabled separate analysis of early preterm deliveries, focus on spontaneous preterm deliveries, and ability to examine a variety of individual- and census tract-level variables. An important limitation was the general nature of the studied markers of social disadvantage; however, it is important to understand contributions of these types of variables, as well as more proximal factors. Several assumptions could impact the validity of our results, including those related to identifiability; although we do not believe our results to be directly causal, we do believe it is important to discuss these assumptions [10]. With such a large sample size, violation of the positivity assumption was not a major concern, but we were careful not to extrapolate beyond levels of variables that were observed among blacks and whites (the positivity assumption refers to the assumption of non-zero probability of observations across combined strata of variables of interest). Temporality is straightforward, in that social disadvantage likely existed before pregnancy began. Residual confounding by social disadvantage certainly may still exist, given the complexity and challenge of measuring it (and thus the assumption of exchangeability may be violated) [28]. We were interested in the overall association with social disadvantage; accordingly, we did not adjust for maternal demographic or health-related characteristics, under the assumption that social disadvantage may have preceded them. This assumption may not be completely valid; for example, although social disadvantage may affect a mother’s age at first birth or parity decisions, her age and parity also affect her level of social (dis)advantage and where she chooses to live. As a case in point, we did not adjust for maternal age at delivery because we considered it to be a potential mediator of the association of interest; however, we also ran models that included age, since it could also be conceptualized as a potential confounder. The ORs for education became modestly larger after adjustment for age (by 0.1–0.2 units for most of the ORs), and the ORs for the other variables in the models changed even less (<0.01 units). This indicates that leaving age out of the models did not substantially influence the overall message of our results. We do, however,encourage further studies that explicitly focus on the potentially complex inter-relationships of social disadvantage and the sociodemographics of childbearing, and their potential joint impacts on disparities. Our ability to assess the stability assumption (i.e., an individual’s exposure-outcome combination is not affect by that of others) is limited. Another limitation is that the generalizability of our results to women with missing data, women having twins or higher order births, and Hispanic women (who comprise almost half of all California births and warrant independent study) is uncertain. California births represent 13% of all U.S. births, but generalizability beyond California births, for example to populations where blacks comprise a larger percentage of all births, is uncertain. We did not incorporate paternal-related variables such as race-ethnicity and education because they were much more likely to be missing for blacks than whites (6% of whites and 22% of blacks were missing father's education, and 4% of whites and 17% of blacks were missing paternal race-ethnicity).

In summary, this study found that the black-white disparity in spontaneous preterm delivery was much more marked for early than moderate preterm deliveries, suggesting that early preterm delivery is a particularly important target for future research on understanding the disparity. We also found that while several of the studied markers of social disadvantage did contribute to the odds of preterm delivery, they tended to have modest potential impact on the disparity, suggesting that future studies should examine more specific factors. Health disparities reflect group differences in health outcomes that are systematic and driven by factors that are potentially remediable [29]; the challenge is to identify these factors, which we expect may improve not just the black-white disparity but also the health of all infants.
